# Pivotal Role of TGF-β/Smad Signaling in Cardiac Fibrosis: Non-coding RNAs as Effectual Players

**DOI:** 10.3389/fcvm.2020.588347

**Published:** 2021-01-25

**Authors:** Somayeh Saadat, Mahdi Noureddini, Maryam Mahjoubin-Tehran, Sina Nazemi, Layla Shojaie, Michael Aschner, Behnaz Maleki, Mohammad Abbasi-kolli, Hasan Rajabi Moghadam, Behrang Alani, Hamed Mirzaei

**Affiliations:** ^1^Physiology Research Centre, Kashan University of Medical Sciences, Kashan, Iran; ^2^Department of Medical Biotechnology, Faculty of Medicine, Mashhad University of Medical Sciences, Mashhad, Iran; ^3^Vascular and Thorax Surgery Research Center, Shiraz University of Medical Sciences, Shiraz, Iran; ^4^Department of Medicine, Research Center for Liver Diseases, Keck School of Medicine, University of Southern California, Los Angeles, CA, United States; ^5^Department of Molecular Pharmacology, Albert Einstein College of Medicine, Bronx, NY, United States; ^6^Department of Medical Genetics, Faculty of Medical Sciences, Tarbiat Modares University, Tehran, Iran; ^7^Department of Cardiology, Faculty of Medicine, Kashan University of Medical Sciences, Kashan, Iran; ^8^Department of Applied Cell Sciences, Faculty of Medicine, Kashan University of Medical Sciences, Kashan, Iran; ^9^Research Center for Biochemistry and Nutrition in Metabolic Diseases, Institute for Basic Sciences, Kashan University of Medical Sciences, Kashan, Iran

**Keywords:** cardiac fibrosis, non-coding RNAs, Smad, TGF—transforming growth factor, microRNA

## Abstract

Unintended cardiac fibroblast proliferation in many pathophysiological heart conditions, known as cardiac fibrosis, results in pooling of extracellular matrix (ECM) proteins in the heart muscle. Transforming growth factor β (TGF-β) as a pivotal cytokine/growth factor stimulates fibroblasts and hastens ECM production in injured tissues. The TGF-β receptor is a heterodimeric receptor complex on the plasma membrane, made up from TGF-β type I, as well as type II receptors, giving rise to Smad2 and Smad3 transcription factors phosphorylation upon canonical signaling. Phosphorylated Smad2, Smad3, and cytoplasmic Smad4 intercommunicate to transfer the signal to the nucleus, culminating in provoked gene transcription. Additionally, TGF-β receptor complex activation starts up non-canonical signaling that lead to the mitogen-stimulated protein kinase cascade activation, inducing p38, JNK1/2 (c-Jun NH2-terminal kinase 1/2), and ERK1/2 (extracellular signal–regulated kinase 1/2) signaling. TGF-β not only activates fibroblasts and stimulates them to differentiate into myofibroblasts, which produce ECM proteins, but also promotes fibroblast proliferation. Non-coding RNAs (ncRNAs) are important regulators of numerous pathways along with cellular procedures. MicroRNAs and circular long ncRNAs, combined with long ncRNAs, are capable of affecting TGF-β/Smad signaling, leading to cardiac fibrosis. More comprehensive knowledge based on these processes may bring about new diagnostic and therapeutic approaches for different cardiac disorders.

## Introduction

Excessive aggregation of ECM, which is mainly produced by myofibroblasts, results in fibrosis (1). In addition, α-smooth muscle actin (α-SMA), a highly contractile protein, is expressed by myofibroblasts. ECM deposition is shown to be reversible, and improved cardiac function and coronary flow result in a minor collagen volume fraction regression (20% relative change and 1% absolute change) (2), an important indicator of ECM content. Additionally, patients with heart failure (HF) are commonly treated with renin–angiotensin–aldosterone system modulators, which lessen cardiac fibrosis (3, 4). Cardiac fibrosis induces pathological processes, which lead to chamber dilatation, muscular hypertrophy, and apoptosis, eventually developing into congestive HF (5). Cardiac fibrosis pathogenesis is complex with no efficient treatment options (6).

Transforming growth factor β1 (TGF-β1) is the principal isoform of TGF-β in cardiac tissue, which can cause Smad2/Smad3 (its downstream mediator) phosphorylation, which in turn can stimulate cardiac fibrosis development. It has been shown in mice that cardiac fibrosis related to pressure overload can be diminished by specific deletion of TGF-β1 or Smad3 gene in the triggered cardiac fibroblasts (CFs) (7). Non-coding RNAs (ncRNAs) include small microRNAs (miRNAs or miRs; > ~22 nucleotides) and long non-coding RNAs (lncRNAs; > ~200 nucleotides), as well as circular RNAs (circRNAs; > ~200 circular nucleotides) (1), all of which are involved in regulating several signaling pathways, including TGF-β and Smad, for the control of cytokine release, along with ECM production (8–10). Evidence corroborates the existence of cross-regulation between the two ncRNAs mediated fibrosis-stimulating pathways and its role in cardiac fibrosis pathophysiology. Recognizing mechanisms associated with such cross-regulation provides possibilities for the development of new therapeutic approaches to reverse cardiac fibrosis (10–12). The present review examines TGF-β, as well as Smad signaling, followed by their contribution in the cardiac fibrosis pathogenesis. In addition, evidence regarding TGF-β and Smad signaling involvement in vascular and cardiac remodeling across fibrotic events is detailed. Finally, ncRNAs (consisting of miRNAs, lncRNAs, and circRNAs) roles in TGF-β and Smad signaling in the heart are discussed. Specifically, the review will focus on the role of TGF-β/Smad signaling in ECM overproduction, cardiac fibrotic event, and myofibroblast alterations, which is the aim of this study. We point out the impacts of miRNAs and lncRNAs, as well as circular lncRNAs, on cardiac fibrosis *via* interaction with the signaling pathways of TGF-β/Smad.

## Pathogenesis of Cardiac Fibrosis

Cardiac fibrosis, namely, the accumulation of scar tissue in the heart, is a product of mismatch between production and degradation of ECM and is strongly associated with cardiac and endocrine disorders (13). Upon stimulation, circulation and myocardial fibrosis–promoting growth factors as well as cytokines levels will increase and initiate a fibrotic response (14). Attachment of the fibrotic-promoting growth factors and cytokines takes place in the corresponding receptors in fibroblasts, namely, cytokine receptors, integrins, syndecans, and CD44 (15), after which signaling pathways and transcriptional factors, such as Smad, mitogen-stimulated protein kinases (MAPKs), nuclear factor κB, and protein kinase B (also called AKT), are activated. These activations induce CFs to transform into myofibroblasts, capable of expressing the strongly contractile protein α-SMA and producing certain tissue inhibitor of metalloproteinases (TIMPs), as well as matrix metalloproteinases (MMPs) for the modulation of ECM homeostasis (14). Additionally, synthesis and release of fibrotic-promoting growth factors and cytokines in CFs are controlled by these transcriptional factors (16). The growth factors and cytokines secreted *via* CFs or other cells, such as cardiomyocytes, and endothelial cells affect CFs or cardiomyocytes and create a positive feedback with final enhancement of the fibrotic responses (16).

In addition to various cell types (such as inflammatory, epithelial, endothelial, and other cells) that contribute to fibrogenesis, three cellular signaling transduction pathways contribute significantly during fibrosis: MAPKs, TGF-β, and integrins. The first pathway, which includes c-Jun NH2-terminal kinase (JNK), p38 MAPK, and extracellular signal–modulated kinase in mammals, has mediating effects on signaling, initiated by extracellular stimulation, such as growth factors and cytokines, or stimulation within the cells (17). The second pathway contributes significantly to the regulation of cellular functions, such as proliferation, differentiation, apoptosis, and survival. Integrins include subunits of α and β, which surface receptors on every cell type with the exception of red blood cells (18). Alongside extracellular receptors, signals transducing pathways engaged in fibrogenesis are triggered by integrins working in coordination with integrin-associated kinases within the cells (18–21).

## TGF-β/SMAD Signaling in Cardiac Fibrosis

**Figure 1:**
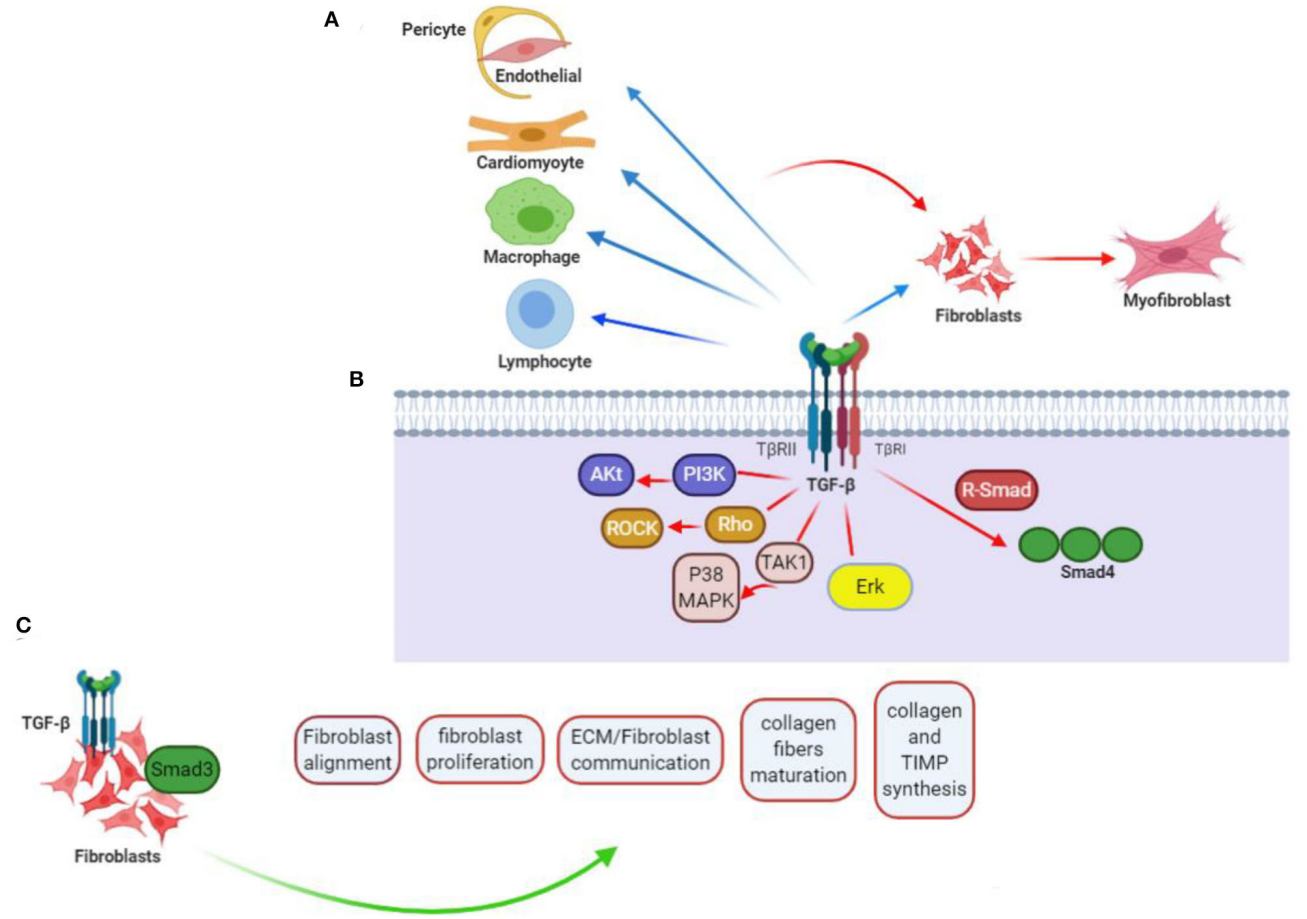
TGF-β contribution to cardiac fibrotic events. **(A)** TGF-β affects phenotype along with operation in every cell somehow engaged in myocardial fibrotic event. Straightforward effects on conversing fibroblast (F) to myofibroblast (MF) and activating myofibroblast can be probably of more importance, but fibrogenesis caused by TGF-βs can further relate with its impacts on the phenotype of macrophage (Ma), differentiation and function of lymphocyte (L), and cardiomyocyte (CM) viability, as well as gene expression. Apart from that, TGF-β can encourage pericyte (P) to fibroblast transformation and endothelial-to-mesenchymal transdifferentiation, when induced by vascular cells to express fibrosis-associated genes. **(B)** TGF-βs regulate phenotypes of the cells through activation of Smad-related together with non-Smad signaling pathways. **(C)** TGF-β/Smad3 signaling effect on CFs. Current researches taking advantage of loss-of-function procedures associated with specific cells drew conclusion that activating Smad3 contributes significantly to the formation of organized myofibroblast arrays after myocardial infarction. Lack of Smad3 in fibroblasts deranges infarcted heart reparations, resulting in higher risks of late cardiac rupture and undesirable chamber dilation. The evidence points out the reparative operation of fibroblasts activated in the infarcted myocardium. Mediation of the Smad3 effects is being carried out by the integrin–ROS axis arousal. This figure was adapted from Frangogiannis (15).

TGF-β can be described as a cytokine with multifunctionality, whose expression takes place by various kinds of cells (22). The superfamily of TGF-β included the TGF-β isoforms (TGF-β1, TGF-β2, and TGF-β3) and activins, as well as inhibins, growth-differentiating factors, bone morphogenetic proteins (BMPs), together with anti-müllerian hormones (AMH) as suborders (23, 24). TGF-β plays a role in different diseases such as cardiac abnormality, cardiac fibrosis, failure of the heart, and remodeling of chamber, as well as cardiac hypertrophy (22) (Figure 1). TGF-β isoforms function with activins toward stimulating signals within the cells through Smad2/3 transcribing factors (25). TGF-β ligand complex has seven different type I receptors (which are sometimes called activin-like kinase or ALK receptors) or five type II receptors (ActRIIA, ActRIIB, TGFBRII, BMPRII, and AMHRII) (26, 27).

It has been previously demonstrated that TGF-β-stimulated clone 22 (TSC-22) could facilitate TGF-β signaling by antagonizing Smad7 activity secondary to enhanced receptor stability. TSC-22 increases TGF-β-induced transcriptional responsiveness and phosphorylation of Smad2/3 (28). Furthermore, the stimulatory effect of TSC-22 is Smad7-dependent, and silencing the expression of Smad7 abolishes TSC-22's effect. TSC-22 can interact with TβRI (TGF-β type I receptor) and Smad7 and prevent the Smad7/Smurfs and TβRI association and receptor degradation. TSC-22 also promotes cardiac myofibroblast differentiation by increasing fibrotic gene expression for α-SMA, fibronectin, plasminogen activator inhibitor 1 (PAI-1), and collagen I, consistent with TSC-22 upregulation and phospho-Smad2/3 in myocardial fibrotic hearts. Therefore, it has been suggested that TSC-22 could regulate TGF-β signaling through a positive-feedback mechanism and may lead to myocardial fibrosis (28).

Binding of type II receptor TGFBRII with TGF-β1 ligands leads to phosphorylation of the type I receptor ALK-5. Various ligands may bind to cell surface TGF-β receptors, which lead to activation of signaling effectors and the Sma- and Mad-related proteins (Smads), as well as interacting with deoxyribonucleic acid (29). TGF-β, myostatin, or activin activates both Smad2 and Smad3, whereas activation of Smad1, Smad5, and Smad8 is performed with BMPs, leading to interactions with Smad4, bringing forth modulating the target gene expression (24, 30, 31). It is noteworthy that TGF-β pathway activation will lead to upregulation of Smad6 and Smad7 expression as well, in turn deactivating the pathways (29). Several ncRNAs and their substrates play a role in the TGF-β signal transduction pathway regulation (21).

Smad2/3 activation affects various profibrotic gene expression, consisting of collagens [COL1A1, COL3A1, COL5A2, COL6A1, COL6A3, COL7A1, (32)], PAI-1 (33, 34), various proteoglycans (35–37), integrins (38), connective tissue growth factor (CTGF) (39), and MMPs (27, 40).

Considerable increase in the levels of TGF-β was observed in individuals experiencing ischemic cardiomyopathy (ICM) and dilated cardiomyopathy (DCM), showing that TGF-β levels correlate with phosphorylated Smad2, along with collagen types I and III, triggering further myocardial fibrotic events in ICM and DCM secondary to activation of TGF-β (41). Fibulin 2 is an essential ECM protein for TGF-β/Smad signaling. Moreover, phosphorylation of Smad2 is achieved only in the presence of fibulin-2 (42). Peroxisome proliferator–activated receptor γ (PPARγ) activation was thought to moderate cardiac fibrosis. A study showed that TGF-β1 directly suppresses PPARγ expression by increasing binding of Smad2/3, Smad4, histone deacetylase 1 (HDAC1), and decreasing binding of HDAC3 to the PPARγ promoter in CFs (43). Another study has shown that reactive oxygen species (ROS) derived from NADPH oxidase 4 (Nox4) enhanced myocardial fibroblasts reaction against TGF-β1 through TGF-β Smad signaling pathways (44). Wnt/β-catenin pathway in inflammatory DCM has been shown to be activated by secretion of Wnt proteins in response to TGF-β signaling, mediated by Smad-independent TGF-β-activated kinase 1 (TAK1) (45, 46). Wnt inactivation or Wnt secretion hindrance impeded TGF-β-mediated CF transformation into pathogenic myofibroblasts, making Wnt protein secretion a neoteric downstream process of TGF-β-modulated cardiac fibrotic development (46). It has been demonstrated that CTGF, also known as CCN2, may play roles in the hypertension-induced myocardial fibrosis through regulation of TGF-β expression (22, 47).

## ncRNAs in Cardiac Fibrosis

ncRNAs are short RNAs that act as epigenetic regulators (48). The regulation of these molecules is related to modulation of several physiological properties such as apoptosis, cell proliferation, metabolism, and differentiation. Deregulation of these molecules shows associations with the onset and progress of various diseases, such as cardiovascular diseases, diabetes, cancer, and inflammatory disorders (49). According to existing evidence, ncRNAs can be categorized into two main groups: (i) short ncRNAs possessing fewer than 200 small nucleotides in their length (i.e., snoRNAs, siRNA, piwi-RNA, and miRNAs), (ii) lncRNAs possessing more than 200 nucleotides in their length including lncRNAs and circRNAs (16, 50). Cardiac fibrosis is a common feature in many types of heart diseases. ncRNA deregulation has been posited to be associated with cardiac fibrosis development and occurrence (49). Table 1 summarizes the role of different ncRNAs contributing to cardiac fibrosis pathogenesis.

**Table 1:** ncRNAs contributing to cardiac fibrosis.

**Non-coding RNAs**	**Effect (s)**	**Expression in CF**	**Targets**	**Signaling pathway**	**Model**	**References**
**miRNA**						
miR-21	Profibrosis	Upregulated	Spry1, PTEN CADM1	↑TGF-β1 → ↓PTEN → ↑MMP-2 CADM1/STAT3 pathway ↑cardiac fibrosis	Rat CFs	(51, 52)
miR-26a/b	Profibrosis	Upregulated	TRPC3	↑MiR-26a → ↓ TRPC3 → ↑CF	Dog fibroblasts model	(53)
			Col1a2/CTGF	miR-26b-5p → ↓Col1a2/CTGF → ↑CF	Mouse CFs	(54)
miR-34	Profibrosis	Upregulated	VEGF, neurogenic locus notch homolog protein 1, vinculin, PPP1R10	Contributing to cardiomyocyte aging; inhibiting miR-34 and limiting cardiac fibrotic events	MI and TAC mice	(19, 55)
miR-132	Antifibrosis	Downregulated	Ras/Rap/SynGAP; methyl-CpG-binding protein 2	Akt/eNOS/Bcl-2 signaling pathway ↓Ras/Rap GTPase-activating protein ↓SynGAP;methyl-CpG-binding domain protein 2 → ↓CF	MI -CD1 mice	(56)
miR-133/miR-30	Antifibrosis	Downregulated	CTGF	Contributing to the progress of fibrosis *via* connective tissue growth factor targeting	Renin-2 tg rat	(57)
miR-133a	Antifibrosis	Downregulated	Collagen α-1(I) chain	Transgenic overexpression in cardiomyocytes inhibits fibrotic progress across overload of pressure and diabetic cardiomyopathy	TAC mice	(58)
miR-155	Profibrosis	Upregulated	Son of seven less gene (Sos1)	Macrophage-derived mir-155–comprising exosomes suppressing proliferation Of Fibroblasts and enhancing inflammation of fibroblasts across cardiac injury	mir-155–deficient mice	(59)
miR-199b	Antifibrosis	Downregulated	Dyrk1a calcineurin/NFAT target gene	Nuclear kinase Dyrk1a is targeted by miRNA-199b in an auto-amplification loop enhancing calcineurin/NFAT signaling inhibition → ↓CF	mouse and human heart failure	(60)
miR-208	Profibrosis	Upregulated	Myosin-6, myosin-7	Inhibition results in decreased progress of fibrosis subject to cardiac stress	miR-208 mutant animals	(61)
miR-214	Antifibrosis	Downregulated	Sodium/calcium exchanger (1Ncx1)	Inhibition results in excessive progress of cardiac fibrosis following myocardial infarction	Ischemic cardiac tissue	(62)
miR-455	Antifibrosis	Downregulated	collagen I and III CTGF	miR-455 → ↓ collagen I and III /CTGF ↓CF	Male diabetic mice	(63)
miRNA-155	Profibrosis	Upregulated	Ski SnoN	↓Antifibrotic Sloan–Kettering Institute proto-oncogene (Ski)/Ski-associated new gene, non–Alu-comprising (SnoN) signaling (negative TGF-β signaling regulating factors) → ↑CF	Diabetic (db/db) mice	(13)
miR−223	Profibrosis	Upregulated	RASA1(RAS p21 protein activator 1)	siRASA1 enhanced MEK1/2, ERK1/2 and AKT phosphorylation → ↑ collagen I, collagen III, and α-SMA → ↑CF	CFs	(64)
miR-9	Antifibrosis	Downregulated	TGFBR2	Suppressing TGF-β receptor II → ↓CF	High glucose/human CFs	(65)
Let-7i	Antifibrosis	Downregulated	IL-6 Mac-2	Let-7i → ↓ interleukin-6/collagens → ↓CF	AngII/mouse; NRCFs	(66)
Let-7c	Antifibrosis	Downregulated	Activate Oct4 and Sox2	Improvement in cardiac function ↓apoptosis, ↓fibrosis, ↓number of discoidin domain receptor 2–positive fibroblasts	MI/mouse; NRCFs	(67)
**lncRNAs**						
lncRNA H19	Profibrosis	Upregulated	ERK1/2, Dual-specificity phosphatase 5 (DUSP5)	↑H19 → ↓DUSP5 (negative regulation of prohypertrophic signaling by↓ ERK1/2) → ↑α-SMA↑/cardiac fibroblast proliferation	Isolated rat cardiac fibroblasts	(68)
			miR-455 CTGF, collagen I, III, α-SMA	H19 and miR-455 modulated myocardial extracellular matrix accumulation	Male diabetic mice	(63)
lncRNA MIAT	Profibrosis	Upregulated	miRNAs-29, 21, 133, 30, and 24	MIAT↑ → miR-24↓ → Furin/TGF-β1↑ → cardiac fibrotic event↑	Anesthesia of healthy male C57BL/6 mice was carried out with Avertin (160 mg/kg, i.p. Sigma–Aldrich)	(69)
Malat1	Profibrosis	Upregulated	miR-145	↑MALAT1 → ↓miRNA-145 (miR-145) → ↑ TGF-β1 → ↑CF	MI mouse heart and AngII-treated CFs	(70)
			Mir-24 Mir-29 Mir-30 Mir-133	↑MALAT1 → ↓ miR-24 → ↑ Furin and ↑TGF-β1 → ↑CF	Mouse model of MI	(69, 71)
Meg3	Profibrosis	Upregulated	p53 signaling MMP-2	Blockage of inducing Mmp-2 expression through TGF r-βI took place with Meg3 silencing by inhibiting P53 binding on the Mmp-2promoter	*In vivo In vitro*	(72)
lncRNA SRA1	Profibrosis	Upregulated	miR-148b	lncRNA SRA1 → ↓ miR-148b → ↑CF	Rat model	(73)
Wisper	Antifibrosis	Upregulated	Splicing of Plod2 mRNA by enabling nuclear localization of TIAR	Regulates cardiac fibrosis after injury ↓Pathological progress of cardiac fibrosis in response to MI while preventing unfavorable remodeling	Murine model of MI	(74)
AK081284	Profibrosis	Upregulated	TGF-β1	IL-17/AK081284/TGF-β1 signaling pathways mediate collagen production → ↑CF induced by high glucose	Diabetic mouse Myocardial fibrosis model	(75)
lncRNA-NR024118 and Cdkn1c	Antifibrosis	Proregulated	↓cell cycle ↓ Cdkn1c	↑ AngII → blocking AT1 receptor → ↓NR024118 → ↑CF	AngII/adult rat CFs	(75, 76)
lncRNA PFL (NONMMUT02255)	Profibrosis	Upregulated	let-7d Ptafr	lncRNA PFL → ↓ let-7d → Ptafr → ↑CF	MI mice cardiac fibrosis in mice	(77, 78)
lncRNA-NONMMUT022554	Profibrosis	Upregulated	ECM–receptor PI3K-Akt	May affect ECM-receptor interactions and the phosphoinositid-3 kinase/protein kinase B (PI3K-Akt) signaling pathway → ↑CF	MI/mouse	(79)
Mhrt	Antifibrosis	Downregulated	Brg1—chromatin remodeling	Binding of Mhrt to the helicase domain of Brg1, a domain which seems critical for tethering Brg1 to chromatin zed DNA targets	Pressure-overloaded hearts by trans aortic constriction	(80)
**Circular RNAs**						
CircActa2	Profibrosis	Upregulated	miR-548f-5p. NRG-1	NRG-1/circACTA2/miR-548f-5p Axis.	Animal model of cardiac remodeling and heart failure	(81, 82)
circAmotl1	Antifibrosis	Downregulation	AKT1/PDK1	↓Dox/↑resistant fibrosis cardiac repair	Cardiac fibroblasts	(83)
circRNA_010567	Profibrosis	Upregulation	↓ miR-141 TGF-β1	CircRNA_010567 → ↓miR-141 → ↑TGF-β1 → ↑Col I, Col III and α-SMA → ↑CF	Mice myocardial fibrosis models	(84)

### miRNAs

As mentioned previously, miRNAs can be defined as short ncRNAs with a length of 18 to 24 nucleotides (85, 86). miRNAs are capable of regulating the function of proteins by binding to target messenger RNA. This may result in the induction of mRNA degradation and/or suppression of protein translation. It has been shown that these molecules modulate myocardial fibrosis pathogenesis (Table 1) (87). Cardiac fibrosis is a complicated process involving the concerted interaction of multiple miRNAs. In this respect, different miRNAs are related to same pathologically fibrotic process. For instance, miR-24, miR-21, miR-34a, miR-29, and miR-433 contribute to fibrosis following infarction, and miR-26a, miR-21, and miR-125b are associated with pressure-overload fibrosis, which is caused by transverse aortic constriction (88–91). In addition, various miRNAs could be classified into antifibrotic (e.g., miR-15 family, miR-101a, miR-145, miR-378, miR-122, miR-142-3p) or profibrotic miRNAs (e.g., miR-29, miR-21, miR-34, miR-208, miR-155, miR-223) (88–91). miRNAs exert their regulatory effects on cardiac fibrosis, although affecting a sequence of cellular and molecular pathways, such as TGF-β/Smad system, MRTF/SRF axis RhoA/ROCK cascade, Wnt signaling, AngII/MAPK signaling, and the cationic channels that regulate calcium responses (92). Callis et al. evaluated miR-208a role in cardiac fibrosis induction. They indicated that miR-208a plays its role *via* targeting THRAP-1 and myostatin in myocardial hypertrophy (93). Furthermore, they showed miR-208a can induce cardiac fibrosis through increased endogen expression (93). Other study demonstrated that the upregulation of miR-208b is related to myocardial function enhancement and could inhibit type I collagen and alias α-SMA. In agreement, miR-208b exerts protection against post-infarction myocardial fibrosis by targeting GATA4 (94).

TGF-β1 can be associated with collagen secretion and activation in myocardial fibroblasts, which play a role in cardiac fibrosis development with other risk factors (95). Furin can modulate TGF-β activation by targeting AngII (96). Bearing that in mind, furin can exert its functions by TGF-β activation (97). Chen et al. showed that miR-24 downregulation is associated with cardiac infarction. Their findings confirmed that miR-24 exerts its effects by inhibiting TGF-β1 with having impact on furin. TGF-β1 and furin levels were elevated, indicating a critical role of miR-24 deregulation in myocardial fibrotic events following myocardial infarction (98).

### Long Non-coding

Intra-action of the cell death and inflammation to myocardial fibrosis is crucial (99). Pyroptosis, namely, cell death triggered by inflammatory reactions, is described by apoptosis and necrosis (100). Nod-like receptor protein 3 (NLRP3) inflammasome expression in cardiac fibrosis is activated by inflammation; subsequently, it activates the cleaved caspase (101). Recent studies have corroborated the contribution of pyroptosis in myocardial fibrosis pathogenesis (102). Nonetheless, the initiating mechanisms for cardiac fibrosis and fibroblast-derived pyroptosis have yet to be determined. Thus, identification of the pathological mechanisms along with efficient treatment targets of myocardial fibrosis is essential. Growth arrest–specific 5 (GAS5), a lncRNA, whose encoding takes place by the GAS5 gene, has been introduced as a tumor suppressor in variety of cancer types (103). GAS5 contributes critically to cell apoptosis and pyroptosis (104). She et al. (105) identified lncRNA-GAS5 as the initiator of pyroptosis in CFs and cardiac fibrotic events. Upon lipopolysaccharide (LPS) stimulation, they detected ISO-induced CF pyroptosis and myocardial fibrosis. Proteins associated with pyroptosis include caspase 1, NLRP3, and DNMT1, higher in cardiac fibrotic tissues, with reduced GAS5 expression. Furthermore, lncRNA GAS5 overexpression enhances and prevents CF pyroptosis and also decreases the expression of caspase 1 and NLRP3 in CF. Other research showed that treating with DNMT inhibitors, 5-aza-2-deoxycytidine, or downregulating DNMT1 caused an increase in expression of GAS5 by reversing promoter hypermethylation in CF. Notably, it has been shown that DNMT1 methylation of lncRNA GAS5 results in CF pyroptosis when NLRP3 axis is affected, suggesting a novel regulatory mechanism regarding CF pyroptosis subject to LPS stress (105).

RNA component of mitochondrial RNA processing endoribonuclease (RMRP) is known as a lncRNA (106). RMRP forms a distinct ribonucleoprotein complex by interaction with the telomerase reverse transcriptase catalytic subunit, which exhibits the activity of RNA-dependent RNA polymerase and makes double-stranded RNAs, which with getting processed can turn into small interfering RNA (siRNA) (106). Prior work has examined the contribution of RMRP to various cancers, such as in lung cancer, gastric cancer, and glioma (107–109). Additionally, Wang et al. (110) reported that the level of RMRP expression in nucleus pulposus tissues correlates with grade of disc degeneration. Another investigation gas demonstrated that overexpression of RMRP could induce nucleus pulposus cell growth and regulate the ECM expression with targeting miR-206. In a recent study, Greco et al. profiled 83-lncRNA expression in biopsies taken from left ventricle of patients suffering HF and corroborated remarkable upregulation of RMRP in these patients (111). Steinbusch et al. (112) found associations of RMRP with chondrocyte hypertrophy and determined chondrogenic differentiation, proposing the contribution of RMRP to the modulation of the dynamic balance of ECM degradation and synthesis. Zhang et al. (113) explored the biological role and mechanisms behind CF induction by the lncRNA, RNA component of RMRP. The findings showed that RMRP expression in an abdominal aortic banding–treated rat model was upregulated in the presence of myocardial fibrosis. Treatment with AngII enhanced RMRP expression in CFs, whereas RMRP knockdown by small-interfering RNA prevented CF proliferation and differentiation as well as collagen accumulation. Based on these findings, RMRP might regulate miR-613 negatively in CFs. Moreover, it was showed that miR-613 mediates the positive effect of RMRP on activation of CF. Based on the present study, RMRP increased CF activation with serving as a competing endogenous RNA for miR-613. Thus, RMRP may represent as a novel target to prevent or treat cardiac fibrosis (113).

### circRNAs

miR-125b induces fibrotic process and upregulation in CFs, indicating numerous binding sites of miR-125b for circ_LAS1L, with inverse association of their expression in those with acute myocardial infarction (AMI) and CFs. RNA immunoprecipitation (RIP), pull-down, and dual-luciferase reporter gene assay supported direct binding of miR-125b bound to circ_LAS1L (114). Overexpressed Circ_LAS1L led to promotion of the downstream target gene secreted frizzled-associated protein 5 (SFRP5) expressions, while reducing α-SMA, collagen I, and collagen III expression; hindering CF proliferation and migration; and increasing apoptosis. Cotransfection with miR-125b mimics and circ_LAS1L overexpression vector did not show considerable changes. However, cotransfection of SFRP5 siRNA and circ_LAS1L overexpression vector resulted downregulation of SFRP5 expression and upregulation of collagen I, collagen III, and α-SMA, as well as enhancement in proliferation and migration of CFs. Accordingly, circ_LAS1L reduces miR-125b activities through its adsorption, consequently increasing SFRP5 and subsequent regulation of the CFs biological properties. Such results can be regarded as a significant experimental basis for regulating myocardial fibrosis following myocardial infarction. CircRNAs contribute critically to the cardiovascular diseases; however, little research has been done on their effect on the myocardial fibrosis. Sun et al. investigated that circ_LAS1L in those suffering AMI and CFs was downregulated and was capable of direct binding to miR-125b, consequently enhancing the downstream target gene secreted frizzled-related protein 5 (SFRP5) expression, finally repressing CF activating, proliferating, and migrating, along with inducing apoptosis. Thus, it is has been posited that the circ_LAS1L/miR-125b/SFRP5 pathway is capable of modulating the biological characteristics of CF and can contribute vitally to the process of cardiac fibrosis, therefore offering a significant theoretical basis to regulate cardiac fibrotic event following myocardial infarction (114).

Gu et al. (115) explored circRNA expression profile and identified circRNA contributions to myocardial fibrosis. Utilization of TGF-β1 aimed at establishing an *in vitro* cardiac fibrotic model in CFs. CircRNA sequencing unveiled that an overall number of 283 circRNAs was expressed abnormally in fibrotic CFs, of which 79 were experiencing upregulation and 204 receiving downregulation. Alterations in randomly selected circRNA expression could be verified with the use of real-time polymerase chain reaction. Establishment of a circRNA-based competing endogenous RNA network 1,755 nodes and 30,394 edges was followed by module analyses performed with implementation of the plug-in MCODE. Kyoto Encyclopedia of Genes and Genomes pathway enrichment analyses targeted mRNAs, engaging in the top three enriched modules. It was found that these mRNAs were enriched in myocardial fibrosis–associated signaling pathways, namely, the AMPK signaling pathway, TGF-β signaling pathway, MAPK signaling pathway, and PI3K-Akt signaling pathway. The predicted ceRNAs and bioinformatics analysis unveiled the possible contribution of circRNAs in myocardial fibrotic event, providing novel knowledge on the mechanisms and searching for efficient preventive, as well as therapeutic targets for myocardial fibrosis (115).

Based on existing evidence, expression of abnormal circRNA takes place in the cardiac fibrotic process. During promotion of CF activated by TGF-β1 or AngII, marked suppression in circRNA circ_BMP2K and miR-455-3p expression has been observed, along with induction of SUMO1 expression (116). RIP, pull-down assay, and dual-luciferase reporter gene assay, demonstrating direct binding of miR-455-3p to circ_BMP2K and their induction of each other's expression. SUMO1 served as a target gene for miR-455-3p, and circ_BMP2K boosted the miR-455-3p inhibiting on the expression of the SUMO1. According to several studies, both circ_BMP2K and miR-455-3p suppressed expressing α-SMA and types I and III collagen, but SUMO1 increased their expression, and the regulatory impacts of circ_BMP2K and miR-455-3p were reversed by miR-455-3p inhibitors or SUMO1 overexpression. Circ_BMP2K and miR-455-3p reduced CF proliferation and migration, concomitantly inducing their apoptosis; however, SUMO1 effect was the opposite; circ_BMP2K and miR-455-3 upregulation on biological characteristics was reversed by miR-455-3p inhibitors or overexpression of SUMO1. Therefore, circ_BMP2K induces expression of miR-455-3p with subsequent downregulation of SUMO1 expression and ultimately prevents CF activation, growth, and migration (116).

## The Relationship Between ncRNAs and TGF-β/SMAD Signaling in Cardiac Fibrosis

### miRNA and TGF-β/Smad Signaling in Cardiac Fibrosis

**Figure 2:**
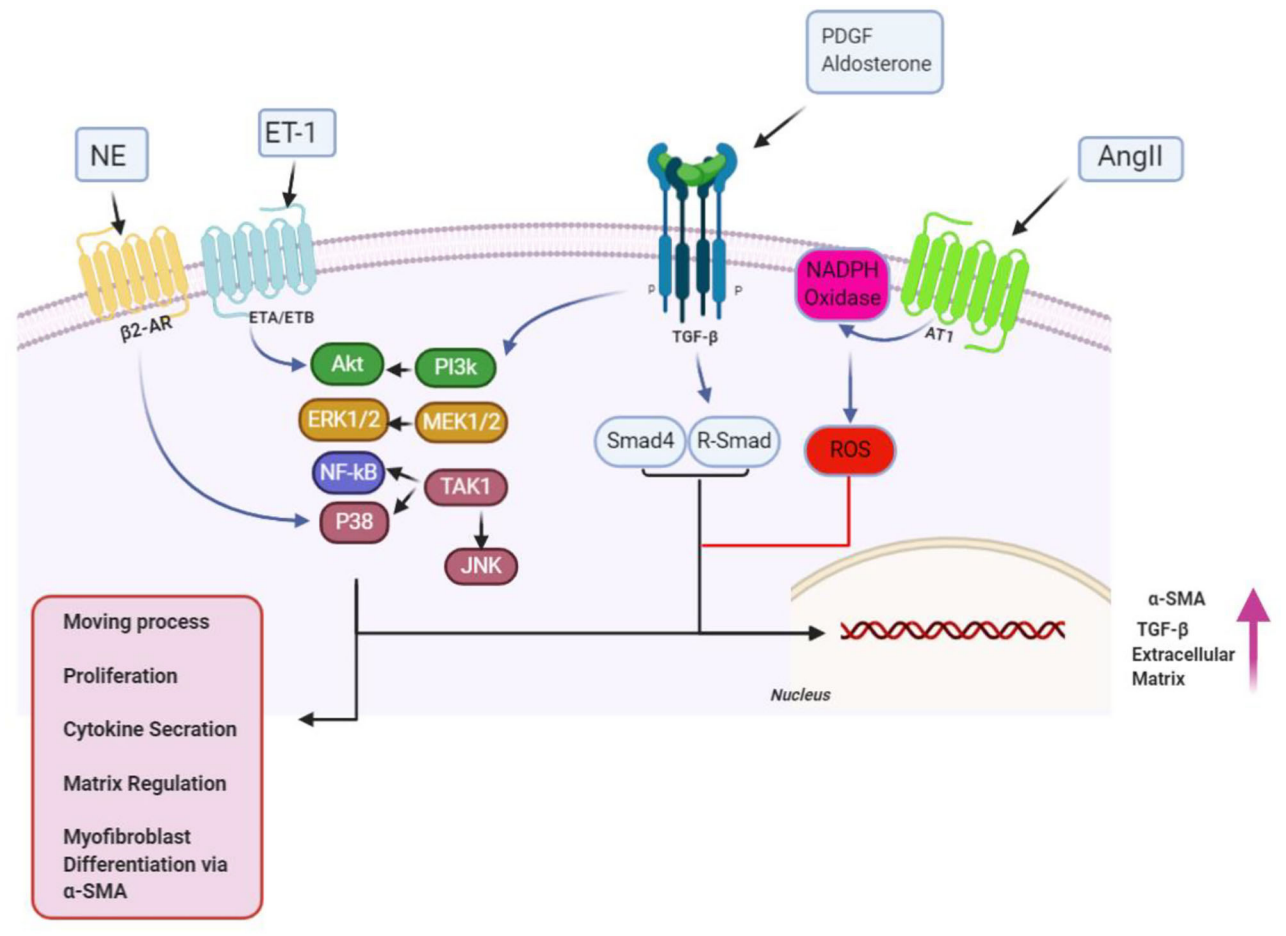
Over the proliferation phase of infarct remedial, fibrogenic growth, as well as neurohumoral mediating factors, stimulates myofibroblast proliferating and migrating, along with activating. A broad scope of fibrogenic mediators, engaged in this phase of cardiac, has been considered in activation of myofibroblast. Neurohumoral mediating factors, including angiotensin II (AngII), aldosterone, and norepinephrine (NE), and growth factors [transforming growth factor (TGF-β), fibroblast growth factor, and platelet-derived growth factor (PDGFs)], together with special matrix proteins, including ED-A fibronectin and matricellular proteins, have cooperation in activating intracellular signaling pathways, which enhance conversion, as well as proliferation of myofibroblast, while modulating ECM protein expression, and of genes related to matrix metabolism. Design of the cartoon took place according to Servier Medical Art (https://smart.servier.com). AR1/4, adrenergicreceptor; ET1/4, endothelin; MMP1/4, matrix metalloproteinase; NF1/4, nuclear factor; ROS1/4, reactive oxygen species 1/4; SMA1/4, smooth muscle actin; TIMP1/4, tissue inhibitor of metalloproteinase. This figure was adapted from Humeres and Frangogiannis (119).

**Figure 3:**
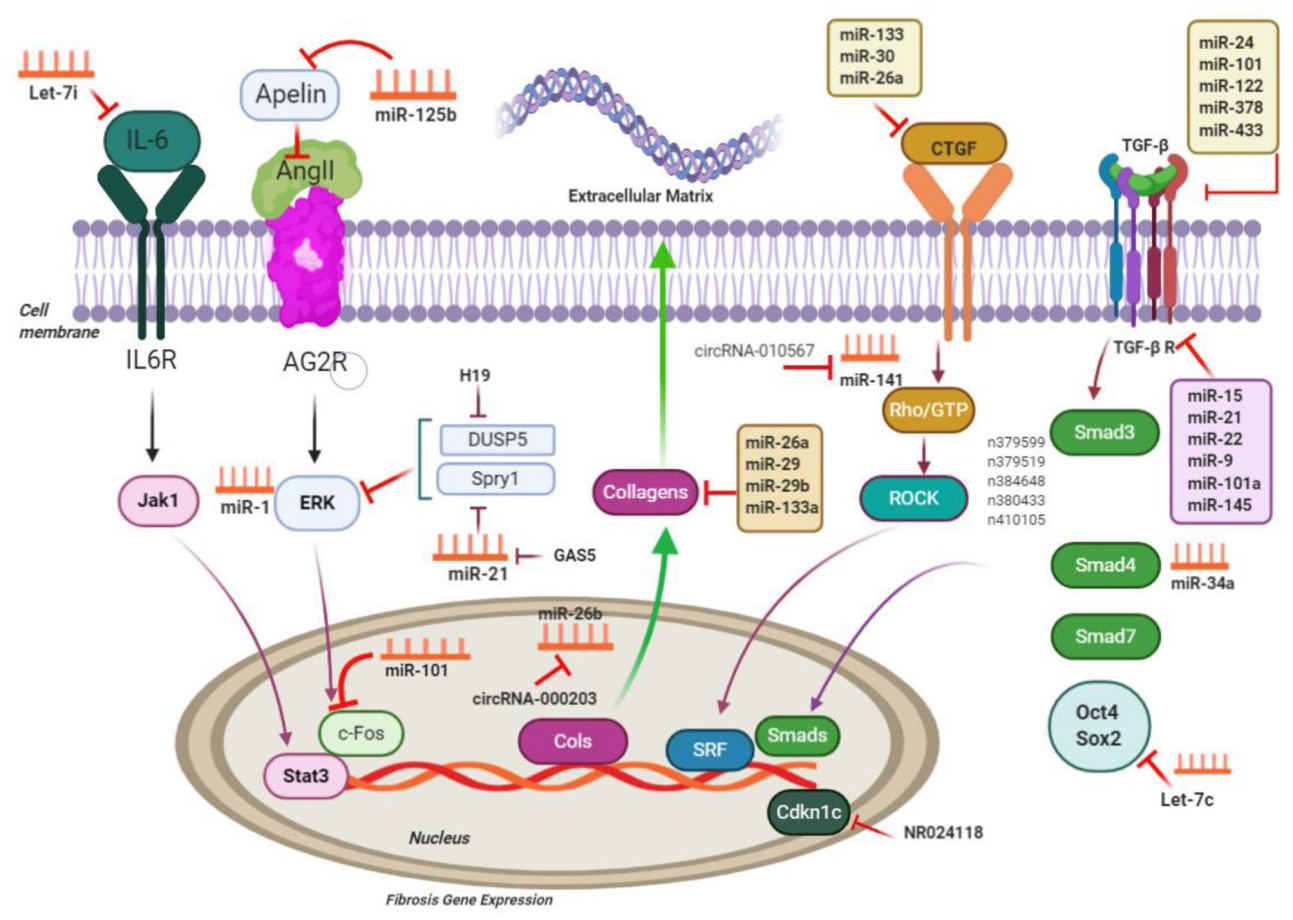
ncRNAs engaged in the pathways of cardiac fibrosis; ncRNAs regulate processes related to cardiac fibrosis *via* targeting the main molecules mediating ECM gene transcription and performing TGF-β signaling; CTGF, connective tissue growth factor; Rho-GTP, Rho-GTPase-stimulating protein; ROCK, Rho related coiled-coil comprising protein kinase; SRF, serum response factor; MMP, matrix metalloproteinases; IL6, interleukin-6; Jak1, Janus kinase 1; Stat3, signal transducers and activators of transcription 3; c-Fos, FBJ murine osteosarcoma viral oncogene homolog; Spry1, sprouty homolog 1; ERK extracellular signal–regulated kinases; DUSP5, dual-specificity phosphatase 5. This figure was adapted from Chen et al. (49).

Various ligands have the ability of binding to TGF-β receptors on the surfaces of cells, permitting regulatory messages transfer to the cells through activation of the signaling effectors, as well as the Sma- and Mad-associated proteins (Smads) and finally, showing interactions with deoxyribonucleic acid (29). Activation of Smad2 and Smad3 are carried out with TGF-β, myostatin, or activin, whereas Smad1, Smad5, and Smad8 are activated by BMPs; activating such proteins leads to interactions with Smad4, resulting in target gene expression modulation (117). Notably, the TGF-β pathway activation additionally leads to upregulation of Smad6 and Smad7 expression, which may end in the pathway deactivation (24, 29). Smad2 and Smad7 lessen fibrosis, but Smad3 results in the promotion of fibrosis (118) (Figure 2). Several miRNAs and their substrates contribute to regulating TGF-β signal transduction pathways (Table 2, Figure 3) (21).

**Table 2:** ncRNAs and TGF-β/Smad signaling in cardiac fibrosis.

**Non-coding RNAs**	**Effect(s)**	**Modulation**	**Targets Smads**	**Signaling pathway**	**Model**	**References**
**miRNA**						
miR-25	Antifibrosis	Downregulated	COL1/COL3 Smad3	miR-25 → ↓ TGF-β1 → ↓collagen I/III	Transaortic constricted mice	(120)
miR-133	Antifibrosis	Downregulated	EP300 COL4A1, FN1 Smad2	↑miR-133a → ↓ phosphorylation of p-ERK1/2 and p- Smad2 → EP300/ ↓ TGF-β1/CTGFL/↓fibronectin/COL4A1 → ↓ cardiac fi/ COL4.	Streptozotocin-induced diabetic in mice	(121)
			Snai1 Gata4, Mef2c, and Tbx5 Mesp1	GMT/miR-133/ Snai1-induced aMHC-GFP → ↑ cardiac reprogramming → ↓CF	Mouse embryonic fibroblasts	(122)
miR-433	Profibrosis	Upregulated	TGF-β1, ERK, p38 kinase, and Smad3	Suppress AZIN1 and JNK1/ /TGF-β1, ERK, p38 kinase, and Smad3 → ↑ cardiac fibrosis.	MI/mice; NRCFs	(123)
miR-29b-3p miR-29c-3p	Antifibrosis	Upregulated	TGF-β2, Mmp2	miR-29b/miR29c → ↑ MIF → ↓ COL1A1, COL3A1/α-SMA,/ Smad3 → ↓ cardiac fibrosis.	(AngII)-infused mouse myocardium Mif-knockout (Mif-KO) mice	(124)
			mRNA 3′-UTR Col 1a1, Col 5a3, and Col 4a2 Smad3	↓TGF-β /Smad3 → ↓collagen I, III, fibronectin → ↓CF	AngII-triggered cardiac fibrotic event in mice Mouse CFs	(125)
			Fibrillins and elastin	Altered the secretion of growth factors and cytokines, including MMP, IGF-1, LIF, and PTX-3 ↓ TGF-β → ↓CFs	AngII (1.46 mg/kg/d, 14 d)-infused mouse myocardium	(20)
miR-21	Profibrosis	Upregulated	↓ Smad7	↑miR21 → ↑TGF-β1 → ↑myocardial fibrosis by inhibiting Smad7	Fibro TAC/mouse	(126)
			↓Smad2/3 ↓ TGF-β R III//p-Smad3	Activate sprouty homolog ↑1/ERK-MAP kinase ↑TGF-β1/Smad2/3 signaling pathway → ↑CF	MI/mouse	(127)
			↑PTEN Spry1	Activate osteopontin/PTEN and ↓Smad7 → ↑CF	AngII/mousesis	(79)
miR-19a-3p/19b-3p	Antifibrosis	Upregulation	TGF-β R II	miR-19a-3p/19b-3p → ↓ TGF-β → ↓ phosphorylation of Smad2 and Akt → ↓CF	Human Cardiac Fibroblasts (HCF)	(128)
miR-24	Antifibrosis	Upregulated	Furin–TGF-β pathway.	↓ TGF-β-p → ↓ Smad2/3 → ↓ Furin → ↓ col-1/α-SMA → ↓CF	Mouse model of MI	(129)
			Smad2/3	↓ TGF-β-p → ↓ Smad2/3 → ↓CF	Mouse model of MI	(130)
			↓JP2(junctophilin-2)	miR-24 regulates excitation-contraction (E-C) coupling by targeting JP2	Aortic stenosis rat model	(131)
miR-26a	Profibrosis	Upregulated	Col1α2, Col1a1	Regulation of nuclear factor nuclear factor κB and progress of fibrosis	AngII/NRCFs	(129)
			CTGF/Smad1	BMP/Smad1 signaling	TAC/IkBa tg mouse	(132)
miR-15 family six miRs (miR-15a, miR-15b, miR-16, miR-195, miR-497, miR-322)	Antifibrosis	Upregulated	↓TGF-βR I	↓ TGF-β pathway ↓ Cardiac remodeling and fibrosis ↑cardiac function	Adult mice under ischemia–reperfusion (I/R) injuries	(133)
			p38, endoglin, Smad3/7	↓ECM remodeling in the overloaded heart ↓ TGF-β pathway	TAC/mouse	(134)
miR-1	Antifibrosis	Downregulated	↓Smad3	↓ TGF-β pathway → ↓ Smad3 → ↓ CF	Mouse models of AngII-induced hypertension	(125)
miR-1	Antifibrosis	Downregulated	Fibullin	Activate ↑fibullin-2/MAPK → ↓CF	AAB/rat	(135)
miR-101a	Antifibrosis	Downregulated	c-Fos Smad3	miR-101 → ↓c-Fos/TGF-β1 pathway → ↓p-Smad3 → ↓CF	Healthy male Sprague–Dawley rats (weight, 200–250 g) and C57BL/6 Mice	(136)
			TGF-βR1	↓TGF-βR I ↓ MAPK → ↓CF	AngII, MI/rat MI, hypoxia/rat NRCFs and MI rat	(137)
miR-34a	Profibrosis	Upregulated	Smad4	↑TGF-β1/Smad4	MI, male C57BL/6 mice (12 weeks of age and a weight of 25–30 g)	
			Suppress PNUTS	Age-triggered expression of miR-34a → ↓PNUTS → inducing DNA damage responses along with telomere attrition → ↑CF	Aging, MI/mice, human	(138)
miR-122	Antifibrosis	Downregulated	Smad4↓	↓TGF-β1 → ↓CF	AS (aortic stenosis patients)/human	(139, 140)
miR-378	Antifibrosis	Downregulated	↓Grb2/TGF /pSmad2/3, IGF1 receptor↓ Activate RTK Integrin β3↓ ↓cFos, ↓c-Jun and Ras	miR-378 → ↓TGF-β1–dependent paracrine mechanisms → ↓fibroblast migration and differentiation	AngII, TAC/mouse; NRCFs	(141)
miR-208a	Profibrosis	Upregulated	↑Smad3/4, ↑endoglin ↑β-MHC	↑miR-208a → ↑TGF-β1 → ↑ endoglin/collagen I → ↑ CF	Aortacaval shunt/rat TAC mouse and RCFs	(142)
			↓Thrap1, myostatin ↑ Endoglin	Induced cardiac fibrosis and cardiac fiand card proliferation	TG mouse	(143)
miR-145	Antifibrosis	Upregulated	TGF-βR II	miR145 acts toward suppression of TGF-β-dependent extracellular matrix accumulation as well as fibrosis	Smooth muscle cells	(144)
			Smad2	Smad2 Alters macrophage sensitivity to TGF-β	AngII/mouse	(145)
miR-125b	Profibrosis	Upregulated	↓Apelin, p53	miR-125b → Inhibition of p53 → induces fibroblast proliferation	TAC, AngII/mouse	(146)
miR-22	Profibrosis	Upregulated	Mimecan/osteoglycin (OGN)	miR-22 → ↓ OGN in age-associated cardiac alterations, including cardiac fibrosis	Aging/mouse; NRCFs	(147)
			Smad4 TGF-βR I in CFs	↑ TGF-β1 → ↑ complex (Smad2/3/4) → ↑ CF	MI mice	(148, 149)
miR-142-3p	Antifibrosis	Downregulated	HMGB1 Smad3	miR-142-3p/HMGB1 → ↓ TGF-β1/Smad3 → ↓apoptosis and fibrosis	Mouse cardiomyocyte M6200 cells received treatment with H/R	
miR-433	Profibrosis	Upregulated	AZIN1 JNK1 Smad3	↓ AZIN1 → ↑ TGF-β1 → ↑ CF ↓JNK1 → ↑ MAPK kinase (ERK/P38) → ↑ Smad3 → ↑ CF	Neonatal rat CFs (8-week-old male C57BL/6 mice)	(123)
miR-499	Profibrosis	Upregulated	Acta1, Smads, Fos, Egr1, Egr2	↑ MAPK kinase (ERK/P38) /↑ TGF-β1 → ↑CF	Neonatal rat cardiac fibroblasts. (NRCFs)	(143)
miR-10a	Profibrosis	Upregulated	↑Collagen I, collagen III, α-SMA, ↓ Smad7	TGF-β1/Smads ↑ Hydroxyproline → ↑cardiac fibrosis and cardiac fibroblast proliferation	Atrial fibrillation (AF) rat	(8)
**lncRNAs**						
lncRNA, Crnde	Antifibrosis	Downregulated	Acta2 α-SMA Smad3	Smad3 → ↑ Crnde → ↑rSBEs → ↓ Binding of Smad3 to the Acta2 / α-SMA gene promoter → ↓CF ↑ Cardiac function	Mouse neonatal cardiac	(150)
GAS5	Antifibrosis	Downregulated	↓ miR-21/PTEN/MMP-2	GAS5 → ↓ miR-21 → ↓TGF-β1/Smad2/3 → ↓CF	ISO/rat; TGF-β1/NRCFs	(151)
5 lncRNAs(n379599, n379519, n384648, n380433, and n410105)	Profibrosis	Upregulated	↑P-Smad2/3 ↑Elastin, periostin, PAI-1, Snai1, Snai2, FBN1	TGF-β pathway → ↑cardiac fibrosis	Ischemic cardiomyopathy	
			Col8A1,Col3A1 fibronection	TGF-β pathway (PAI-1, Snai1, Snai2,/p-Smad2/3) → ↑cardiac fibrosis	ICM/human; mouse CFs	
lncRNAs CHRF	Profibrosis	Upregulated	miR-489	CHRF → regulate MyD88 and Smad3 by targeting miR-489 → ↑CF	AngII-treated myocytes Mouse model Human heart failure samples	(81, 152, 153)
**Circular RNAs**						
circ_000203	Profibrosis	Upregulated	MiR-26b-5p BMP/Smad1	CircRNA_000203 → ↓ miR-26b-5p(anti-fibrotic) → ↑ Col1a2 /Col3a1/α-SMA CTGF → ↑CF BMP/SMAD1 signaling	AngII/mouse CFs Diabetic mouse myocardium	(54, 84, 129, 154)
CircRNA_010567	Profibrosis	Upregulated	↓miR141 TGF-β/Smad pathway	CircRNA_010567 → ↓miR-141 → ↑ TGF-β1 → ↑Col I/ Col III/α-SMA. → ↑CF	Diabetic mouse Myocardial fibrosis model	
circRNA–circNFIB	Antifibrosis	Upregulated	miR-433 TGF-β/Smad3	↑circNFIB → ↓miR-433 → ↓CF	Mice post-MI cardiac fibroblasts	
circHIPK3	Pro fibrosis	Upregulated	miR-29b-3p Smad3	circHIPK3 → ↓miR-29b-3p → ↑ TGF-β/Smad3 → ↑α-SMA, COL1A1, COL3A1 → ↑CF	AngII-induced cardiac fibrosis	(155)

miRNA-associated TGF-β pathways in cardiac fibrotic event exert their effects when they target the common ECM protein CTGF. Moreover, it was demonstrated that miR-101 inhibited interstitial fibrosis and then, by inhibition of a c-Fos/TGF-β1 axis, may promote myocardial infarction (136). Downregulation of miR-101 was evident in infarcted myocardium in mice and in angiotensin-cultured CFs. Interestingly, miR-101 overexpression inhibited proliferating and producing COL through suppression of its target c-Fos and the downstream protein TGF-β1 (136). Transfection of miR-101 mimic significantly suppressed the expression of TGF-β RI and p-Smad3, CF differentiation, and collagen content (137). According to He et al., miR-21 may reinforce the TGF-β1/Smad signaling pathway in atrial fibrosis stimulated by AF, through Smad7 downregulation (126). A reciprocal loop was ascertained between miR-21 and its target TGF receptor III, causing ECM remodeling and fibrotic process. Upregulation of cardiac miR-21 occurred in infarcted myocardium as a result of TGF-β1/Smad2/3 signaling pathway activation, whereas downregulation of its target gene (TGF receptor III) was evident. Nevertheless, lower expression of the TGF receptor III reinforced TGF-β1/Smad2/3 signaling pathway (126, 156).

Thum et al. (127) showed promotion of myocardial fibrosis by miR-21 by targeting extracellular modulated kinase inhibitor sprouty homolog 1 (Spry1) while activating MAPK signaling in cardiac fibroblasts. In a myocardial ischemia–reperfusion model, miR-21 was found to target Pten, subsequently leading to an increase in matrix metalloprotease 2 (Mmp2). Consistently, miR-21 antagonism leads to increased Pten in cardiac fibroblasts (157). miR-24 overexpression reduced secretion of the TGF-β and phosphorylation of the Smad2/3 in CFs (130). miR-24 showed protective features against myocardial fibrosis following myocardial infarction, which was dependent on the inhibitive effects on its target gene FURIN, suppressing the TGF-β signaling pathways (98, 130). Wang et al. (130) demonstrated interference of miR-24 with TGF signaling by targeting the pro-protein convertase, furin, and then downregulation of TGF level in cardiac fibroblasts with targeting CTGF. miR-18a and miR-19b downregulated the expression of the collagen (COL) 1A1 as well as COL3A1, reducing cardiac fibrosis in age-related cardiac failure triggered through activation of TGF-β (19, 124, 128). Functional examinations are consistent with prevention of HCF autophagy by miR-19a-3p/19b-3p with targeting TGF-β R II mRNA. Furthermore, autophagy development releases suppressive effects of miR-19a3p/19b-3p on Smad2 and Akt phosphorylation *via* TGF-βRII signaling (128).

In addition, many other miRNAs were also recognized to target collagens and TGF signaling to contribute to the fiwere also. For example, Let-7i and miR-26a reduce collagen deposition and impose their effects by targeting Col12 and Col11, correspondingly (66, 132, 158). miR-29b upregulation because of TGF/Smad3 inactivation downregulated profibrotic genes, such as ECM genes elastin (159), fibrillin 1 (Fbn1), collagen type I, 1 and 2 (Col11, Col12), and collagen type III, 1 (Col31) (160) and enhanced cardioprotective impacts of carvedilol vs. myocardial fibrosis triggered by AMI (79, 125). It was shown that insulin-like growth factor 1 and leukemia inhibitory factor, which are targeted by miR-29b, play roles in activating CF and proliferating ECM (20, 124).

Tao et al. investigated that miR-433 was related to cardiac fibrosis and is a potential target to mitigate cardiac fibrosis. Their study has found that cardiac fibrosis induces miR-433, subsequently decreasing the expression of AZIN1 and JNK1. Downregulated AZIN1 induces TGF-β1 pathway, whereas decreased JNK1 results in ERK and p38 kinase activation, causing Smad3 activation and eventually leading to cardiac fibrosis (123). In another study in that same year, Ooi et al. (161) suggested that AZIN1 expression reduction induces TGF-β/Smad3 signaling activation in CFs; (III) reduced JNK level would enhance ERK, P38 kinase, and Smad3 phosphorylation, and that is in turn associated with proliferation and differentiation of fibroblast into myofibroblasts.

miR-133a contribution to cardiac fibrosis and electrical repolarization in adult hearts with pressure overload can potentially indicate its regulatory impacts on Col11 A1, Serca2a, and calcineurin expression (58, 162). Based on existing evidence, miR-133a overexpression has prevented myocardial fibrotic event in both AngII-related hypertension and diabetes, even though the effector proteins were different in diabetes (fibronectin and COL4A1) and AngII-related hypertension (COL1A1) (21, 121, 162). Moreover, overexpression of the cardiac miR-133a inhibited ERK1/2 and Smad2 phosphorylation. Accordingly, it is posited that miR-133a may show efficacy in treating myocardial events triggered by diabetes (90, 121).

miR-15 family members are also regarded as having antifibrotic characteristics, through functions against TGF-β-mediated actions (163). miR-15 family members (miR-15a, miR-15b, miR-16, miR-195, miR-497, miR-322) can be observed in a variety of cardiac cell types, and with cardiac stress, they are expressed at higher levels (134, 163, 164). miR-15, in fibroblasts, targets some of TGF-β signaling cascade members, such as TGF-β1, p38, endoglin, Smad3, and Smad7, and as a result, leads to negative regulation of ECM production. Correspondingly, *in vivo* miR-15 suppression with LNA-based anti-miRs in mice resulted in higher levels of fibrosis following transverse aortic constriction (163). However, the miR-15 family inhibition in a mouse model of reperfusion injury led to smaller infarct sizes and lesser cardiac remodeling (134). Table 2 lists various non-coding RNAs in the CF *via* activation/inhibition of Smad/TGF signaling pathway.

### lncRNA and TGF-β/Smad Signaling in Cardiac Fibrosis

Several lncRNAs contribute to the TGF-β pathways affecting the ECM gene expression along with myofibroblast differentiation (165). According to Huang et al., regulation of lncRNAs expression took place in ICM dynamically, in which several lncRNAs further attend in the TGF-pathways provoking gene expression associated with accumulating collagen along with ECM protein encoding genes (e.g., COL14A1, COL16A1, COL12A1, COL8A1) and myofibroblast differentiation. Huang et al. reported altered lncRNA expression in ICM and demonstrated that CF-enriched lncRNAs such as n379599, n379519, n384648, n380433, and n410105 in mouse modulate the fimouse-associated gene expression by targeting TGF-β signaling (165). TGF-β expression targets PAI-1, Snai1, and Snai2 in CF, and several lncRNA overexpression indicated induction of these target gene expression by lncRNAs. It was also demonstrated that lncRNAs induced phosphorylated Smad2/3 and not Smad2/3 protein (165, 166).

Tao et al. recently studied the lncRNA growth arrest–specific 5 (GAS5) role and function in cardiac fibrosis and concluded that GAS5 *via* negative miR-21 regulation plays its suppressive role in cardiac fibrosis. Moreover, they demonstrated that the modulation of miR-21 regulated MMP-2 expression *via* a phosphatase as well as tensin homolog (PTEN) pathway in CFs (151). miR-21 down regulation decreased secretion of TGF-β and phosphorylation of Smad2/3 in CFs (126).

lncRNAs and cardiac fibrosis CHRF (cardiac hypertrophy–related factor) upregulation was noted in myocytes treated with AngII and in the heart of a mouse model with transverse aortic constriction and human HF sample (152). CHRF knockdown increased miR-489 level but decreased Myd88 level in myocytes. CHRT overexpression reduced miR-489 level and upregulated Myd88 level and resulted in hypertrophic responses. Cardiac fibrosis was decreased in Myd88-knockout mice. CHRF regulates MyD88 and Smad3 by targeting miR-489. This study proposed CHRF as a role player in cardiac fibrosis by miR-489 and Myd88 adjustment (81, 152). lncRNA Crnde, by means of Smad3-Crnde negative feedback in diabetic cardiomyopathy, alleviates cardiac fibrosis. Crnde overexpression markedly prevents α-SMA promoter activity induced by TGF-β. Crnde stops Smad3 transcriptional activity *via* rSBEs (RNA SBEs) (49, 150, 165, 166).

### circRNA and TGF-β/Smad Signaling in Cardiac Fibrosis

Zhou et al. (84) showed that circRNA-010567 boosts myocardial fibrosis through suppression of miR-141 suppression along with targeting TGF-β1 in a mice model with diabetes. In another recent article, it was shown that upregulation of CircRNA_000203 took place in diabetic mice cardiac muscle and in AngII-triggered fibroblasts in the animal's heart (54). CircRNA_000203 characterizes as a miR-26-5p sponge and interacts with miR-26-5p and fibrosis-related genes Col1a2, Col3a1, and α-SMA and CTGF in fibroblasts in mouse heart (54, 167).

Zhu et al. suggested that the circNFIB–miR-433 axis can potentially provide new therapeutic target to treat fibrotic diseases. circNFIB overexpression decreased pro-proliferative impacts stimulated by means of the miR-433 mimic, while inhibiting circNFIB led to contrary results. circNFIB was recognized as a miR-433 endogenous sponge. circNFIB upregulation also lessened the activation of p38, ERK kinases, and the Smad3 signaling pathways were indicated through reduced ratios of p-p38/p38, p-ERK/ERK, and p-Smad3/Smad3 (168).

CircHIPK3 expression led to a significant increase in CFs and heart tissues following AngII treatments. CircHIPK3 silencing decreased CFs proliferating as well as migrating and the α-SMA expression level upregulation triggered by AngII *in vitro*. circHIPK3 served as a miR-29b-3p sponge, and circHIPK3 overexpression reversed miR-29b-3p–triggered inhibition of CF proliferation and migration, while altering miR-29b-3p targeting genes (α-SMA, COL1A1, COL3A1) expression levels *in vitro*. circHIPK3 silencing and miR-29b-3p overexpression conjointly exerted more severe effects on suppression of cardiac fibrotic event *in vivo* compared to either of them alone. In addition, the expression of circHIPK3 was also markedly increased after TGF-β1 treatment (155). Their data suggested that circHIPK3 functions as a miR-29b-3p sponge in the adjustment of CF proliferating, migrating, and promoting cardiac fibrotic event, introducing possible novel targets to be explored in preventing cardiac fibrosis triggered by AngII (155).

## Conclusion

The uncompromising progress of fibrosis represents a pathological finding inherent to multiple cardiac diseases. Gaining insight into these fibrotic processes in terms of the functional characteristics and molecular profiling could make it possible to prevent and treat fibrotic lesions in the heart. An enlarging body of evidence addresses the cross-talk between the TGF-β and Smad signaling pathways and its contribution to cardiac fibrosis pathogenesis. Despite the fact that the TGF-β and Smad pathways have been extensively studied, their contributions to profibrotic pathways in cardiac diseases are yet to be known. ncRNAs have been identified as possible role players in strategies for mitigating CVDs, as discussed before. Current research on ncRNAs described herein focuses on the role of ncRNAs in regulating cell signaling pathways, particularly TGF-β and Smad signaling. The identified signaling pathways discussed herein, which have roles in the involvement of ncRNAs in cardiac fibrosis, may offer novel putative targets for therapeutic approaches for cardiac fibrosis. More studies are required to better understand the mechanisms by which the ncRNA network induces cardiac fibrotic events *via* TGF-β/Smad signaling. In addition, the potential clinical significance of the TGF-β/Smad-associated ncRNAs, including miRNAs implemented as therapeutic instruments and circRNAs employed as diagnostic/prognostic biomarkers for cardiac fibrotic cases, needs testing in additional animal models as well as clinical conditions.

## Author Contributions

HM involved in conception, design, statistical analysis, and drafting of the manuscript. LS, SN, MAs, MM-T, SS, BA, MN, BM, MAb, and HR contributed in data collection and manuscript drafting. All authors approved the final version for submission.

## Conflict of Interest

The authors declare that the research was conducted in the absence of any commercial or financial relationships that could be construed as a potential conflict of interest.
